# Accounting for the symmetric and asymmetric effects of FDI-growth nexus amidst financial crises, economic crises and COVID-19 pandemic: application of hidden co-integration

**DOI:** 10.1186/s43093-022-00131-x

**Published:** 2022-06-25

**Authors:** Rowland Tochukwu Obiakor, Kingsley Ikechukwu Okere, Obumneke Bob Muoneke, Nnamdi Chinwendu Nwaeze

**Affiliations:** 1 Department of Economics, Babcock University, Ilishan-Remo, Ogun State Nigeria; 2 Department of Economics, Banking and Finance, Gregory University, Uturu, Abia State Nigeria; 3 Department of Finance, University of Lagos, Akoka-Yaba, Nigeria; 4 Department of Economics, Abia State University, Uturu, Nigeria

**Keywords:** Linear and nonlinear, FDI, Economic crisis, COVID-19, Economic growth, Cobb–Douglas, Nigeria

## Abstract

This study investigates the symmetric and asymmetric effects of FDI-growth nexus amidst financial crises, economic crises and COVID-19 pandemic s in Nigeria over the period 1983–2020. Having confirmed the long-run stable state among the variables, the symmetric estimates suggest that the FDI inflow/outflow is significantly linked with economic growth both in the long and short run, while the asymmetric model suggests that the parameter estimates have asymmetric effects on economic growth during the economic crisis in Nigeria. Specifically, the positive shocks in FDI inflow/outflow generate a significant reducing impact on economic growth, while negative shocks in FDI inflow/outflow generate a significant increasing effect on economic growth. Based on this findings, the study suggests that policy option would need to generate more economic activities that would improve net inflow/outflow and cushion the effect of future crisis.

## Introductory aspect

Asides the devastating impact of the 2007–2008 global financial crisis which shook the Nigerian capital market and other economic fundamentals, focus extends to the recent economic recession orchestrated by the commodity oil price shock in 2016 which led Nigeria to its first recession in decades due to its unrepentant reliance on the black gold as its major source of forex and limiting factor for yearly appropriation. The dire implication of the oil price shock saw a multiplicity of shortages due to a superior benchmark per barrel used in budget planning necessitating governments’ last resort to borrowing from local and international sources to fund budgets, service debts and carry out capital and developmental projects. After extant economic recovery in Q1 2017, growth numbers remained within the threshold of 2–3% correlated with a recovery in the official world price of crude oil. However, there was another dip in the official price of crude oil adversely impacted by the COVID-19 pandemic in 2020. Demand shock is penciled as the chief orchestrator obviously trigged by the COVID-19 pandemic with joint causatives such as reduced economic activity which created an oversupply causing oil prices to plunge downward rapidly. The main reason the effect of crisis is more devastating in developing countries as opposed to advanced economies is the paucity of resources necessary for stimulating the economy and more importantly protecting the vulnerable population from untold hardship culminating from multiplier effect of the crisis. Iqbal [21] in support posited that developing countries are never the root cause of crisis but are the most affected. The author went further to add the resulting implications such as contraction of exports, credit crunch, decline in industrial production, increasing unemployment, external debt crisis, poverty, reduced capital flows and foreign direct investment inflows in particular with devastating multiplier effects on growth and other measures bordering on welfare.

FDI-growth nexus based on linearity is well pronounced in academic literature theoretically and empirically in developing countries (see [11]), however, the scope of this research enlarges the discussion beyond the already established positive linear association between FDI and economic growth especially in the Nigerian case. Specific to our scope is an extensive focus on FDI and growth nexus amidst global financial crisis and other variants of crisis in the case of Nigeria. The justification for the inclusion of FDI is mirrored in the claims of Egboro [14] and Bandara [8] positing that macroeconomic variables such as foreign direct investment, exports and foreign portfolio investment are spearhead recipients of global shocks in the case of Nigeria and Sub-Saharan Africa. In simpler terms, the impact of financial crisis is transmitted through macroeconomic variables on to the nation’s economy, hence the adoption of FDI as the loci in our analysis.

The nexus between FDI-growth amidst crises is established in the study of Jimborean and Kelber [24] where the scholarly duo posited that the global financial crisis adversely affected growth. On the other hand, surprisingly, FDI in the face of financial crisis contributes significantly to economic growth. Convincingly, the scholarly duo established that decline in real GDP growth rates in Central and Eastern European countries during the 2007 financial crisis possesses no adverse effect on FDI- growth link. Contrasting evidence from a study conducted by Breitenlechner et al. [9] courtesy its large panel dataset comprising 67 developing countries opined that in the face of financial crisis, FDI leads to significantly worse economic outcomes. Gaies et al. [18] enlarged the debate further positing that FDI contributes to economic growth in the long run for developing countries and increases it further by diminishing the recessionary effect emanating from a banking crisis. From the stance of the aforementioned studies, we can deduce two standpoints,a) financial crisis transmitting through FDI increases GDP b) financial crisis transmitting through FDI decreases GDP.

Papers in this area are classified into thematic categories which shows the coverage footprint of previous authors on the aforementioned debate; FDI-financial crisis nexus [10, 13, 28, 37–39] and FDI-growth nexus amidst crisis periods which has received few entries which is sub-divided in its crises selection,one crisis period [18] and two crises period [24]. All three studies are focused on Central and Eastern European countries and developing countries leaving a large vacuum in academic literature. On the home-front (Nigeria), there is a scarcity of studies on comprehensive empirical investigation of FDI-growth nexus in crisis periods in the case of Nigeria coupled with massive patronage of linear based approach by previous authors to the FDI-growth nexus debate excluding crisis both in Africa and Diaspora. Our quest beyond the realm of symmetry stems from the standpoint of Amin et al. [5] where nonlinear relationship between fdi outflows and economic growth was established in Romania, reemphasizing that rise and fall in FDI outflows impact economic growth positively in Romania. However, Jimborean and Kelber [24] attempt to examine the FDI-growth nexus amidst global financial crisis and the euro area sovereign debt crisis focused solely on FDI inflows, while other authors utilised aggregate FDI as seen in Dornean et al. [13] and Bandara [8]. There is little or no evidence on the relationship between fdi outflows and growth amidst financial and economic crisis further increasing the novelty of this study encompassing both fdi inflows and fdi outflows to increase the generalization of our projected findings. The purpose of this study is to ascertain if there is presence of symmetric and asymmetric effects in the FDI-growth nexus amidst the global financial crisis, commodity oil price shock and COVID-19 pandemic.

The uniqueness of this empirical adventure and its contribution to knowledge addendum is listed thus; (a) debut study to investigate the presence of symmetric and asymmetric effects in the FDI-growth nexus in aforementioned crisis periods, i.e., 2007–2008 global financial crisis, commodity oil price shock 2016 and COVID-19 pandemic in Nigeria (b) filling the lacuna by adopting a nonlinear approach to the debate employing the nonlinear ARDL with multiple structural breaks to test research hypothesis. (c) decomposition of FDI into net inflows and outflows is required to ascertain the transmission of shocks from crisis to FDI and from FDI to GDP (d) Incorporating relevant crisis periods beyond the popularised global financial crisis used by a multitude of authors globally to increase the generalisability of study findings. The rest of the paper is arranged in chronological order review of related literature, methods and material, empirical result, summary and conclusion.

## Theoretical literature

### Theoretical orientation

There are numerous benefits accruing to FDI inflows in contributing to the growth of host economies as supported by prima-facie empirical evidence (see [4, 42]. Theoretical standpoint reinforced in the study of Moura and Forte [29] outlines some channels through which FDI engenders growth namely, (a) transfer of new technologies and technical know-how (b) human resources (c) integration into the global economy (d) increased competition in host markets (e) economic and political interference. Some group of economists posit that for growth to materialise, the availability of human capital proficient in imbibing technological and knowledge transfers in line with De Mello [12], Ford et al. [16]. Setting the theoretical build-up of this study further, the neoclassical growth theorists,Solow and Swan, indeed major theorists in that school of thought stressed the point of capital accumulation in a bid to avail the productive sectors of necessary finance to carry out productive activities that are growth-inducing in the long term.

Adopting business cycle theory in explaining the nature and movement of FDI amidst financial crisis or other variants of crisis is succinct. Broner et al. [10] affirms that gross capital flows are pro-cyclical in nature prompting the adoption of two states of nature, crisis and non-crisis periods. The latter has been exhausted as it pertains our topic especially using a linearized approach in the case of Nigeria, whereas the former is under-explored necessitating our interest in filling up the lacuna. The interdependence of countries through foreign trade and financial flows quadruples the probability of conveying shocks from the originating country to other countries embedded in the global economic system. Bandara [8] posited that despite a multiplicity of theories explaining the transmission of financial crisis and other economic crises, the ardent scholar classified the significant theories into two,theories explaining fundamental causes and theories linked to investor behavior.

### Empirical literature

As earlier posited in the introductory part of this novel study, the three phases under which empirical posits of various authors will be examined are FDI-financial crisis nexus, FDI-growth nexus and FDI-growth nexus amidst crisis periods and other factors imperative such as trade.

#### FDI-financial crisis nexus

The degree of response of FDI and other portfolio flows is showcased in Uctum and Uctum [39] where the scholarly duo investigated FDI-crisis nexus in the case of Turkey relying on structural breaks and the regression-based approach by Bai and Perron (1998) informed readership that although FDI and FPI react in times of crisis but foreign direct investment reacts more to domestic crisis while foreign portfolio investment reacts more to global financial quagmires. Broner et al. [10] further expanded the discourse stating that capital flows are pro-cyclical positing strongly that the modus operandi is two-way,firstly, during expansions, foreigners invest in host countries abroa, while domestic agents invest outside the shores. Secondly, in the face of crisis, there is a sudden collapse in the total stock of gross capital flows necessitating a retrenchment in the inflows by foreign investors and a corresponding reduction in outflows by domestic agents. Furthermore, the scholarly quadruple empirically confirmed that gross capital flows react more strongly to global financial crises than domestic crises. Ucal et al. [38] contributed more distinctively to the discourse by investigating whether financial crisis influences FDI inflows using a long panel dataset and semiparametric Generalized Partial Linear Model (GPLM) as its sole econometric technique. The authors posited through the auspices of two dummies (Crisis and Crisisc) where the former focuses on year after financial crisis and the latter focuses on a year before the financial crisis, while the zeros remained rooted to the alternative direction. Ucal et al. [38] strongly asserted that FDI inflows dip in the successive years post-financial crisis and a vast reduction in FDI inflows a preceding year prior financial crisis hitting a country. Dornean et al. [13] investigated global financial crisis, economic crisis and FDI flows nexus in the case of CEE countries in the EU. Findings show that economic growth possesses an outstanding influence on FDI, and crisis possesses a negative impact on FDI. Mahmoud [28] examined the impact of financial crises on bilateral foreign direct investment (BFDI) using system GMM as its prime econometric technique. Findings are in perfect consonance with a-priori expectations pointing strongly to the established fact that financial crises possess an adverse impact on FDI in host and home countries. A relevant study conducted by Fu et al. [17] provided empirical evidence relating to a global pandemic-fdi nexus. The trio authors more specifically analyze the impact of COVID-19 on FDI margins depending on a global monthly dataset and Heckman two-stage bias selection approach as its econometric technique. The results were specific rather than popular generic findings common in the available literature. The study posited that the impact of COVID-19 on extensive margin and intensive margin translates to investors unwillingness to invest in a country where the virus is spreading rapidly. Furthermore, the authors documented that higher casualties in host country reduce the FDI value it can attract by a significant magnitude. Focusing on existing FDI, COVID-19 had minimal impact, also the completion of FDI transactions is met with severe delays when the home country is plagued with many cases. Fang et al. [15] added to the COVID-19 and foreign direct investment nexus in OECD, BRICS and emerging countries. Several measures were adopted to measure the impact of COVID including number of new cases, new deaths, cumulative cases, cumulative deaths, and active cases. Findings show that the numbers of new confirmed cases, new deaths, and cumulative confirmed cases are found to have significant negative impacts on FDI in the case of OECD, BRICS and emerging countries.

#### FDI-growth nexus

Under this tranche, studies executed on the aforementioned topic; foreign direct investment and economic growth nexus are reviewed simultaneously.

Ehigiamusoe and Lean (2019) investigated the foreign capital inflows-growth nexus in the case of Nigeria using ARDL-ECM framework. The scholarly duo recorded the positive effect of foreign portfolio investment on growth, whereas foreign direct investment and foreign aid possess no significant impact on growth signalling that Nigeria’s economic growth is not sustainable in as much as reliance on FDI and foreign aid is maintained. The state of academic literature on this topic is divided as some of the prima-facie studies in the last two decades reports negative effect and positive effects in their various studies which is influenced by influx of FDI inflows into Nigeria’s telecom sector, majority of FDI inflows directed to the oil and gas sector and the model estimated vis-à-vis econometric tools utilised. Akinlo [3] offered ample empirical evidence as regards the sectoral location of FDI inflow into Nigeria using the traditional OLS framework. The revered scholar posited that foreign capital possesses a minute impact on economic growth, however, the small effect is due to the gross inability of extractive FDI to induce growth. Ayanwale [7] followed suit going deeper on the sectoral FDI distribution to investigate the empirical relationship between non-extractive FDI and economic growth in Nigeria relying on OLS and two-stage least squares as its prime econometric technique. Findings proved worse than Akinlo [3] stating that overall FDI effect on growth is insignificant, however, sectoral FDI has a positive impact on growth which in this case is the FDI in telecommunication sector possessing the superior potential to grow the Nigerian economy as opposed to oil sector and manufacturing FDI. Going further to the close of the decade, Adegbite and Ayadi [2] relying on traditional OLS and robust checks posited strongly showcasing the beneficial effect of FDI on economic growth in Nigeria, however, is limited by the level of human capital development and infrastructural development. Acquah and Muazu [1] provided ample evidence in the case of West Africa on the same debate. The scholarly duo opined that the varying positive effect of FDI on growth is largely dependent on the model specification, however, it was found that higher levels of FDI are associated with higher levels of growth which coincides with the case of Nigeria. Furthermore, Awolusi and Adeyeye [6] relying on traditional OLS and Generalized Method of Moments posited that FDI’s impact on economic growth in Africa and Nigeria in specific remains minute and unsubstantial in consonance with Akinlo [3] and Ayanwale [7]. Udi et al. [40] begged to differ using traditional ARDL positing that FDI inflows contribute more significantly to economic expansion of the Sub-Saharan region compared to trade openness and exchange rate. Yeboua [41] further deepened empirical insights involving financial development in the FDI-growth nexus using panel smooth transition regression model (PSTR) as its main econometric tool. Findings shows that there is a minimal financial development threshold level above which in African nations, the growth-enhancing effect of FDI is unlocked. Iqbal et al. [23] added more spice to academic literature by investigating the impact of the Belt Road Initiative project on the growth of Asian economies. The far-reaching impact of the BRI outside the shores of Asia increases its relevance in the FDI-growth nexus amidst crisis discourse. Findings from the study posited that BRI wields a significant impact on growth of Asian economies. Qualitative factors influencing the flow of FDI in the case of China and India are seen in the study of Hasan et al. [19] as corruption was fingered to possess a negative effect on FDI in the case of India. On the other hand, corruption was found to possess a positive impact on FDI in the case of China. Iqbal and Rahman [22] posited that asides FDI been a major culminator of growth, SMEs contribute significantly to economic growth in ASEAN economies same with the Nigerian case.

#### FDI-growth nexus amidst crisis

Navigating to studies that have investigated the FDI-growth nexus amidst crisis, extant search shows that the few empirical entries made are fully focused on Central Europe, Eastern Europe and a panel of developing countries (lower and middle-income countries). Jimborean and Kelber [24] investigated the fdi-growth nexus amidst the global financial crisis and the euro sovereign debt crisis in the case of CEE countries relying on panel data GMM estimator (two-step) and fixed-effect panel estimator. Findings revealed that global financial crisis possessed a negative impact on FDI inflows and GDP, while the 2011 euro area crisis further worsened growth rates and net fdi inflows in the case of Central and Eastern European countries. The final empirical entry on the fdi-growth nexus amidst financial crisis was provided by Gaies et al. [18] taking a larger sample of 67 developing countries comprising lower and middle-income economies and adopting two-step system GMM and panel logit model as its econometric technique. In line with the findings of Jimborean and Kelber [24], foreign direct investment retained its positive effect on growth in line with the a-priori expectations, on the other hand, foreign direct investment increases growth by reducing the recessionary effect of crisis on growth up to a critical threshold, thereafter, the relationship turns negative. Nwosa [31] in the case of Nigeria investigated the performance of dire macroeconomic indicators amidst the global pandemic. The author depending on daily data from December, 2019 to May, 2020 and VAR causality econometric technique concluded that the COVID-19 pandemic adversely affected oil price, exchange rate and stock market performance in Nigeria, which in turn has devastating multiplier effects on growth. Furthermore, Nwosa [31] posited that in comparison 2009 global financial crisis and the recession that shook the Nigerian economy in 2016, COVID-19 pandemic possessed a greater effect on the trilicate (oil price, exchange rate and stock market) as advertised in Okere et al. [32].

## Methods and material

### Description data and sources

Data harnessed for this empirical adventure owe origin from the World Bank Development indicators, and the statistical publications of the CBN spanning between 1983 and 2020 are employed in the current study. The justification for this time frame is the readiness of data series from the reliable sources. The data for this study include FDI inflows % of GDP, export and import % of GDP, FDI outflows % of GDP, economic crisis-dummy variable encapsulating financial crises, economic crises and COVID-19 pandemic, real capital stock, domestic credit to private sector to GDP and trade openness to GDP, economic growth as real gross domestic product.

#### Model specification and justification of variables

A popular model employed in this study is the neoclassical model where technology, capital stock and labour are germane. In this growth model, factors of production (labour and capital) and technology are the key determinant of growth; hence, there is extant need to believe in the positive association between FDI and Solow residual factor, justifying the empirical compatibility of neoclassical model in study. In achieving the aim of this study, Cobb–Douglas production function is implemented. The production function is expressed thus

1 $$Q_{t} = A_{t} L_{t}^{\beta } K_{t}^{1 - \beta } \quad 0 < \beta < 1$$

In Eq. 1, *Q*, *K*, *L* and *A* are the real output stock, capital stock, labor force, and technological advancement affecting the productivity of *K* and *L*. To achieve the desired objectives-that is, the role of economic crisis in between FDI-economic growth relationship, we argument Eq. 1 in line with the extant studies [1–3] thus a combination target variables (FDI and interaction terms) and control variables. By relaxing the assumption of constant returns to scale and introducing natural logarithm transformation, the model specification is given as

2 $$\begin{aligned} \ln Q_{t} & = \theta_{0} + \theta_{1} \ln L_{t} + \theta_{2} \ln K_{t} + \theta_{3} {\text{Dum}}_{t} + \theta_{4} \ln {\text{FDI}}_{t} \\ & \quad + \theta_{5} \ln \left( {{\text{FDI}}*{\text{Dum}}1} \right)_{t} + \theta_{6} \ln {\text{Trade}}_{t} + \theta_{7} \ln {\text{Pcrd}}_{t} + \varepsilon_{t} \\ \end{aligned}$$

$\ln Q_{t}$ is for real GDP-Real GDP is a sufficient measure of economic growth in our study, although per capita GDP has been used tremendously in empirical literature, our study pitches its tent in Real GDP to capture economic growth.

$\ln {\text{FDI}}_{t} \;{\text{is}}$ FDI inflow/outflow as a % GDP. We have decomposed FDI into inflow and outflow. During crisis, inflows and outflows occur simultaneously in some countries, while in others, it may occur in a one-way direction as pronounced in the available academic literature. Nigeria is a practical example where FDI is highly sensitive to economic policy shifts necessitating outflows and inflows depending on the scenario. Therefore, the two variables will be employed in the two baseline equation. It is expected to be positive, $\theta_{4} > 0$.

${\text{Dum}}1_{t}$: Crisis is dummy variables indicator. We employ a single dummy variable to proxy crises that affected Nigeria throughout the timespan covered namely; 2007–2008 global financial crisis and commodity oil price shock in 2016 and COVID-19 pandemic. We encompassed all the events into one dummy variable, so it takes the value of 1 for 2007–2009, 2016 and 2019 and 0 for otherwise. It is expected to have a negative sign $\theta_{5} < 0$.

${\text{FDI}}*{\text{Dum}}1$: It the interaction term and medium the through which financial crises, economic crises and COVID-19 pandemic impact on economic growth vis-à-vis FDI. The aim of this variable is to capture the conditional effect on FDI on economic growth, given the moderating role of economic crisis. The negative effect indicates the extend FDI would decrease the rate at which its spillover effect is transferred to economic growth.

ln*L* for labor force. This variable is an unchangeable component of the Cobb–Douglas production function and also contributes to output as posited in the study of Ilgun et al. [20] on FDI-growth nexus in the case of Turkey. It expected to positive $\theta_{1} > 0$.

ln*K* is for real capital stock, Real capital stock generated from gross fixed capital formation: Capital accumulation has been tainted by many economists as a channel for engendering growth and vast number of scholars prefer to use gross fixed capital formation to that effect. $\theta_{2} > 0$.

$\ln {\text{Trade}}_{t}$: This trade openness % GDP and expected to be $\theta_{6} > 0$; $\ln \;{\text{pcrd}}_{t}$ is domestic credit to private sector % GDP.$\theta_{7} > 0$ $\varepsilon_{t}$ depicts the white noise error term.

#### Linear and nonlinear ARDL model specifications

An ARDL bound testing approach to co-integration popularized by Pesaran et al. [33] is a frequent model used in the investigation of the causal-effect relationship between endogenous and exogenous. It is reliable and robust when the variables are *I*(0) or (1), even when the exposition are of order mixed *I*(0) and *I*(1). Thus, in the presence of model’s reliability and stability, the long and short run can be estimated accordingly. The baseline model for standard ARDL is shown in Eq. 2 as thus:

3 $$\begin{aligned} \Delta \ln Q_{t} & = \alpha_{0} + \alpha_{1} \ln Q_{t - 1} + \alpha_{2} \ln L_{t - 1} + \alpha_{3} \ln K_{t - 1} + \alpha_{4} \ln {\text{FDI}}_{t - 1} \\ & \quad + \alpha_{5} \ln \left( {{\text{FDI}}*{\text{Dum}}1} \right)_{t - 1} + \alpha_{6} \ln {\text{Trade}}_{t - 1} \\ & \quad + \mathop \sum \limits_{i = 1}^{p} \alpha_{1i} \Delta \ln Q_{t - 1} + \mathop \sum \limits_{i = 1}^{q} \alpha_{2i} \Delta \ln L_{t - 1} + \mathop \sum \limits_{i = 1}^{V} \alpha_{8i} \Delta \ln K_{t - 1} \\ & \quad + \mathop \sum \limits_{i = 1}^{m} \alpha_{4i} \Delta {\text{Dum}}1_{t - 1} + \mathop \sum \limits_{i = 1}^{k} \alpha_{5i} \Delta \ln {\text{FDI}}_{t - 1} \\ & \quad + \mathop \sum \limits_{i = 1}^{d} \alpha_{6i} \Delta \ln \left( {{\text{FDI}}*{\text{Dum}}1} \right)_{t - 1} + \mathop \sum \limits_{i = 1}^{g} \alpha_{7i} \Delta \ln {\text{Trade}}_{t - 1} \\ & \quad + \alpha_{{{\text{Dum}}2}} {\text{TB}}rk_{t} + \varepsilon_{t} \\ \end{aligned}$$

Multiple structural break in model specification is proxied by dum2 where 1 is used for the break point day and 0 for the other days. Lag length in the distributed lag section is $p,q,v,m,k,d\;{\text{and}}\;g$. Next, we checked for the presence of co-integration under the assumption of no cointegration among variables in the null hypothesis, thus, $H_{0} : \alpha_{1} = \alpha_{2} = \alpha_{3} = \alpha_{4} = \alpha_{5} = 0$. Following Narayan, [30], Pesaran et al. [34] modeling protocol, the null hypothesis is rejected if the estimated F-statistics from Eq. (2) is greater than the upper critical limit, accepted if it is less than the lower critical limit, and inconclusive if it is still within the crucial lower limit. Further, the error correction model $\left( {{\text{ecm}}} \right)$ in Eq. 3 describes the dynamics in the short-run exposition from Eq. (2).

4 $$\begin{aligned} \Delta \ln Q_{t} & = \alpha_{0} + \mathop \sum \limits_{i = 1}^{p} \alpha_{1i} \Delta \ln Q_{t - 1} + \mathop \sum \limits_{i = 1}^{q} \alpha_{2i} \Delta \ln L_{t - 1} + \mathop \sum \limits_{i = 1}^{V} \alpha_{8i} \Delta \ln K_{t - 1} \\ & \quad + \mathop \sum \limits_{i = 1}^{m} \alpha_{4i} \Delta {\text{Dum}}1_{t - 1} + \mathop \sum \limits_{i = 1}^{k} \alpha_{5i} \Delta \ln {\text{FDI}}_{t - 1} + \mathop \sum \limits_{i = 1}^{d} \alpha_{6i} \Delta \ln \left( {{\text{FDI}}*{\text{Dum}}} \right)_{t - 1} \\ & \quad + \mathop \sum \limits_{i = 1}^{g} \alpha_{7i} \Delta \ln {\text{Trade}}_{t - 1} + \alpha_{{{\text{Dum}}2}} {\text{TB}}rk_{t} + \lambda_{{{\text{1ecmt}}}} + \varepsilon_{t} \\ \end{aligned}$$

Based on $\lambda_{1}$, the coefficient of ${\text{ecm}}_{t - 1}$, model performance may be judged. Theoretically, if $\lambda_{1}$ turns out to be negative and statistically significant, it would indicate that a probable short-term shock will eventually lead to a long-term equilibrium. Following Shin et al. [36], we extend Eq. 3 & 4 to derive the NARDL model specification by incorporating partial positive and negative sums of the independent variables, thus, decomposing the independent variable FDI inflow/outflow. Implementing NARDL to this study has some scholarly advantages: (1) applicable when *I*(0) and *I*(1) are both presence and included in the model; but, it does not require *I*(2) before it can be implemented. (2) it reveal hidden co-integration by accounting for asymmetric and nonlinear relationship between variables [35].

Next, we present partial positive and negative sums component of FDI shown as thus

5 $$\ln {\text{FDI}}_{t} = \ln {\text{FDI}}_{0} + \ln {\text{FDI}}_{t}^{ + } + \ln {\text{FDI}}_{t}^{ - }$$

where the initial value at time *t* = 0 is $\ln {\text{FDI}}_{0}$. The decomposed partial positive and negative sums for $\ln {\text{FDI}}_{t}$ are $\ln {\text{FDI}}_{t}^{ + }$ and $\ln {\text{FDI}}_{t}^{ - }$ and shown as follows

6 $$\ln {\text{FDI}}_{t}^{ + } = \mathop \sum \limits_{j = 1}^{t} \Delta {\text{FDI}}_{j}^{ + } = \mathop \sum \limits_{j = 1}^{t} = \max \left( {\Delta {\text{FDI}}_{j} ,0} \right)$$

7 $$\ln {\text{FDI}}_{t}^{ - } = \mathop \sum \limits_{j = 1}^{t} \Delta {\text{FDI}}_{j}^{ - } = \mathop \sum \limits_{j = 1}^{t} = \min \left( {\Delta {\text{FDI}}_{j} ,0} \right)$$

The NARDL exposition is shown as

8 $$y_{i} = \rho + \rho^{ + } \ln {\text{FDI}}_{t}^{ + } + \rho^{ - } \ln {\text{FDI}}_{t}^{ - } + \varepsilon_{t}$$

The asymmetric long-run estimates in Eq. (8) are $\rho^{ + }$ and $\rho^{ - }$ in Eq. (8) highlighting the impact of positive and negative changes in $\ln {\text{FDI}}$. Substituting (8) into Eq. (2), the NARDL exposition could be modified as thus

9 $$\begin{aligned} \Delta \ln Q_{t} & = \alpha_{0} + \alpha_{1} \ln Q_{t - 1} + \alpha_{2} \ln L_{t - 1} + \alpha_{3} \ln K_{t - 1} + \alpha_{4}^{ + } \Delta \ln {\text{FDI}}^{ + } + \alpha_{5}^{ - } \Delta \ln {\text{FDI}}^{ - } \\ & \quad + \alpha_{6} \ln \left( {{\text{FDI}}*{\text{Dum}}} \right)_{t - 1} + \alpha_{7} \ln {\text{Trade}}_{t - 1} + \mathop \sum \limits_{i = 1}^{p} \alpha_{1i} \Delta \ln Q_{t - 1} + \mathop \sum \limits_{i = 1}^{q} \alpha_{2i} \Delta \ln L_{t - 1} \\ & \quad + \mathop \sum \limits_{i = 1}^{V} \alpha_{8i} \Delta \ln K_{t - 1} + \mathop \sum \limits_{i = 1}^{\omega } \alpha_{4i}^{ + } \Delta \ln {\text{FDI}}_{1t - 1}^{ + } + \mathop \sum \limits_{i = 1}^{\varphi } \alpha_{5}^{ - } \Delta \ln {\text{FDI}}_{2t - 1}^{ - } + \mathop \sum \limits_{i = 1}^{m} \alpha_{6i} \Delta {\text{Dum}}1_{t - 1} \\ & \quad + \mathop \sum \limits_{i = 1}^{k} \alpha_{7i} \Delta \ln {\text{FDI}}_{t - 1} + \mathop \sum \limits_{i = 1}^{d} \alpha_{8i} \Delta \ln \left( {{\text{FDI}}*{\text{Dum}}} \right)_{t - 1} + \mathop \sum \limits_{i = 1}^{g} \alpha_{8i} \Delta \ln {\text{Trade}}_{t - 1} + \alpha_{{{\text{Dum}}2}} {\text{TB}}rk_{t} + \varepsilon_{t} \\ \end{aligned}$$

where $$\rho^{ + } = {\raise0.7ex\hbox{${\alpha_{4}^{ + } }$} \!\mathord{\left/ {\vphantom {{\alpha_{4}^{ + } } {\alpha_{1} }}}\right.\kern-\nulldelimiterspace} \!\lower0.7ex\hbox{${\alpha_{1} }$}}$$ and $$\rho^{ - } = {\raise0.7ex\hbox{${\alpha_{5}^{ - } }$} \!\mathord{\left/ {\vphantom {{\alpha_{5}^{ - } } {\alpha_{1} }}}\right.\kern-\nulldelimiterspace} \!\lower0.7ex\hbox{${\alpha_{1} }$}}$$ are associated with positive and negative long-run components, while the short-run components are captured by $$\sum\nolimits_{i = 1}^{\omega } {\alpha_{4i}^{ + } \Delta \ln {\text{FDI}}^{ + } }$$ and $$\sum\nolimits_{i = 1}^{\varphi } {\alpha_{5i}^{ - } \Delta \ln {\text{FDI}}^{ - } }$$, respectively. To confirm the long-cointegration, we followed the same protocol as advised by Shin et al. [36] that is line linear ADRL procedure. Accordingly, Eq. (9) produces *F*-statistic required for decision about two hypothesis that is null $$\alpha_{4}^{ + } = \alpha_{5}^{ - } \;{\text{and}}\;{\text{the}}\;{\text{alternative}}\;{\text{as}}\; \alpha_{4}^{ + } \ne \alpha_{5}^{ - }$$. To maintain single hypothesis, F-statistic and critical value are estimated from Eq. (9) with respect to the extreme and lower bounds. In line with Narayan [30], null hypothesis is rejected, if F-statistic supersedes the upper critical bound, it implies asymmetric effect is validated, if F-statistic is less than the lower bound, it is considered as inconclusive, if F-statistics exits within the lower and upper critical bounds, asymmetric effect is not validated.

There are three categories of asymmetry in a model: adjustment, short-run and the long-run asymmetry. Asymmetries in long- and short-run time horizons are studied using the Wald test, which is distributed as $$\chi^{2}$$ with one degree of freedom [36] and must be significantly different as thus $$\rho^{ + } \ne {\raise0.7ex\hbox{${\alpha_{4}^{ + } }$} \!\mathord{\left/ {\vphantom {{\alpha_{4}^{ + } } {\alpha_{1} }}}\right.\kern-\nulldelimiterspace} \!\lower0.7ex\hbox{${\alpha_{1} }$}}$$ and $$\rho^{ - } \ne {\raise0.7ex\hbox{${\alpha_{5}^{ - } }$} \!\mathord{\left/ {\vphantom {{\alpha_{5}^{ - } } {\alpha_{1} }}}\right.\kern-\nulldelimiterspace} \!\lower0.7ex\hbox{${\alpha_{1} }$}}$$.$$\sum\nolimits_{i = 1}^{\varphi } {\alpha_{7i} \Delta \ln {\text{FDI}}^{ + } } = \sum\nolimits_{i = 1}^{\omega } {\alpha_{7i} \Delta \ln {\text{FDI}}^{ - } }$$, respectively, while the adjustment asymmetry involves demodulate the asymmetric cumulative dynamic multiplier on $$\ln Q_{t}$$ for unit changes in $$\ln {\text{FDI}}^{ + }$$ + and $$\ln {\text{FDI}}^{ - }$$ using the following equations:

Extraction of asymmetric cumulative dynamic multiplier using the following equation:

10 $$m_{h}^{ + } = \mathop \sum \limits_{j = 0}^{h} \frac{{\partial \ln Q_{t + j} }}{{\partial \ln {\text{FDI}}_{t}^{ + } }} ,m_{h}^{ - } = \mathop \sum \limits_{j = 0}^{h} \frac{{\partial \ln Q_{t + j} }}{{\partial \ln {\text{FDI}}_{t}^{ - } }} ,h = 0,1,2$$

If $$h \to \infty ,$$ then $$m_{h}^{ + } \to \rho^{ + }$$ and $$m_{h}^{ - } \to \rho^{ - }$$ where $$\rho^{ + } = {\raise0.7ex\hbox{${\alpha_{4}^{ + } }$} \!\mathord{\left/ {\vphantom {{\alpha_{4}^{ + } } {\alpha_{1} }}}\right.\kern-\nulldelimiterspace} \!\lower0.7ex\hbox{${\alpha_{1} }$}}$$ and $$\rho^{ - } = {\raise0.7ex\hbox{${\alpha_{5}^{ - } }$} \!\mathord{\left/ {\vphantom {{\alpha_{5}^{ - } } {\alpha_{1} }}}\right.\kern-\nulldelimiterspace} \!\lower0.7ex\hbox{${\alpha_{1} }$}}$$ are the asymmetric coefficient.

## Empirical result, interpretation and discussion of findings

### Preliminary analysis

The partial correlation is shown in Table 1. For example, there is a negative correlation between FDI-inflow/outflow and economic growth. Other variables have a significant positive correlation with economic growth except labor. The data series were subjected to three types of stationarity tests with the view to checking that none of the variables is *I*(2), and thus check for the reliability of F-statistics. The first duo are augmented Dickey-Fuller (ADF) and Philips-Perron (PP) tests that do not account for policy or structural shift, while the third test, Lee and Stazicich [26], account for multiple breaks and captures policy or structural shift. In Table 1, ADF and PP test reveal that FDI-inflow and outflow at level are found to be stationary, while other variables at first difference attained stationarity. In Table 2, expanding the discourse on stationarity position, Lee and Stazicich [26] test reveals two possible structural breaks in two regimes, suggesting that FDI-inflow and outflow are attained stationarity at level, while the other at first difference. The justification for the structural break in the Nigerian economy is mirrored through the implementation of series of industrial policies, bank recapitalization exercise and economic crisis in 2000 & 2010 leading to shocks in the FDI. In all, none of the variables is *I*(2), that makes this study a candidate of linear and non-ARDL model.

**Table 1:** ADF and PP test without structural break, and correlation matrix

Level form	ln*Q*(− 1)	Lninflow	lnoutflow	ln*L*	ln*K*	lnPcrd	lntrade
ADF	− 0.4421 [1]	− 3.9591 [0]^a^	− 3.1772 [7]^b^	0.9317 [6]	− 2.4650 [9]	− 0.5581 [3]	− 2.6056 [0]
PP	− 0.1587 [3]	− 3.8436 [4]^a^	− 4.0979 [3]^a^	− 0.9059 [4]	− 1.2391 [2]	− 1.0788 [11]	− 2.5962 [6]
First difference							
ADF	− 4.2391 [0]^a^	− 4.3372 [0]^a^	− 4.6936 [9]^a^	− 3.3567 [5]^b^	− 4.4272 [0]^a^	− 5.3631 [2]^a^	− 4.0890 [0]^a^
PP	− 4.3221 [2]^b^	− 12.86 [29]^a^	− 11.263 [6]^a^	− 7.5521 [3]^a^	− 4.4011 [9]^a^	− 9.1237 [33]^a^	− 7.4488 [0]^a^
Correlation matrix							
*Q*	1.0000						
Inflow	− 0.0356	1.0000					
Outflow	− 0.1533	0.6567	1.0000				
* L*	− 0.7642	0.1435	0.0548	1.0000			
* K*	0.6457	− 0.2868	− 0.3713	− 0.3722	1.0000		
Pcrd	0.7869	0.1051	− 0.1429	− 0.5107	0.4772	1.0000	
Trade	0.1568	0.2807	0.1822	0.0602	− 0.2244	0.2134	1.0000

^a,b^Indicate 1% and 5% level of significance

**Table 2:** Lee and Stazicich [26] unit root test with structural break

Variables	Level form *I*(0)		First difference *I*(1)		Results
	t-statistics	Break points	t-statistics	Break points	
lnQ	− 4.5913 [6]	2000–2010	− 4.3056 [8]	2000–2007	*I*(1)
lninflow	− 11.118 [6]^a^	1994–2009	− 9.1377 [5]^a^	1993–1999	*I*(0)
lnoutflow	− 11.826 [5]^a^	1995–2003	− 15.425 [7]^a^	1994–2004	*I*(0)
ln*L*	− 9.5718 [8]^a^	2003–2011	− 11.637 [1]^a^	1993–2010	*I*(0)
ln*K*	− 4.6835 [4]	1996–2004	− 6.5194 [2]^b^	2006–2012	*I*(1)
lnPcrd	− 3.4053 [2]	2005–2012	− 8.8050 [7]^a^	2004–2007	*I*(1)
lntrade	− 7.2328 [6]^a^	1994–2003	− 8.0427 [6]^a^	2002–2014	*I*(0)

^a,b^Indicate 1% and 5% level of significance

### Test for co-integration

In view of the order of the integration, ARDL bound testing method is implemented. Studies in co-integration are prone to lag sensitivity and to ameliorate this econometric challenge, the initial step is to choose the optimal lag selection using the Akaike information criterion (AIC). Lag 2 is proposal based on AIC as the optimal for the bound test. ${\text{DumTBrk}}$ is mirrored on the industrial policies, bank recapitalization exercise and economic crisis. Tables 3 and 4 are used in this study to examine the asymmetric relationship between economic growth and FDI during the financial crisis by looking at the F-statistics for the two baseline model specifications of the linear and nonlinear ARDL model. FDI inflows and outflows are broken down into a positive and negative partial sum. Nigeria’s long-run relationship between the variables has been confirmed by the rejection of the null hypothesis of no co-integration. Stability and validity of the parameters were checked by CUSUM and CUSUMSQ recursive residuals, while the validity of the regression output was confirmed by Durbin-Watson stat, Jarque–Bera normality test, BG serial correlation LM test, Heteroskedasticity test: ARCH and Ramsey RESET test. Indeed, the findings of this research can be used in a variety of ways to help shape and analyze policy.

**Table 3:** FDI-inflow for symmetric and asymmetric ADRL bounds co-integration test

Table. ARDL bounds cointegration test results	Best ARDL model	F-statistic	Result
Specifications	ARDL(1, 0, 0, 1, 2, 1, 2, 0,0)	6.1032^a^	Cointegration
1. *F*_*Q*_ (ln*Q*|ln*K*, ln*L*, lnDum1, lninflow, $\ln \left( {{\text{in}} {\text{flow}}*{\text{Dum}}1} \right)$*,* lnPcrd, lnTrade, Brk)			
Diagnostic test			
D–W	2.6319		
JB- normality test	0.5286	[0.7677]	
LM test	8.1523	[0.0170]	
Het: ARCH	17.493	[0.2308]	
RESET test	0.4703	[0.5016]	

Cusum & Cusumsq stable		I(0) Bound	I(1) Bound
Critical value bounds (*k* = 8)	10%	2.196	3.37
	5%	2.597	3.907
	1%	3.599	5.23
2. *F*_*Q*_ (ln*Q*|ln*K*, ln*L* lnDum1, lninflow^+^, lninflow^−^, ln*(*inflow*Dum1), lnPcrd, lnTrade, Brk)	ARDL(2, 2, 2, 2, 2, 2, 1, 2, 1, 1)	6.4246^a^	Cointegration
Diagnostic test			
D–W	2.524487		
JB- normality test	0.751	[0.6869]	
LM test	4.2948	[0.1168]	
Het: ARCH	6.189	[0.9974]	
RESET test	1.0474	[ 0.3248]	

Cusum & Cusumsq stable		I(0) Bound	I(1) Bound
Critical value bounds (*k* = 9)	10%	1.8	2.8
	5%	2.04	2.08
	1%	2.5	3.68
The model selection ARDL is based on (AIC)			
a,b & c indicate level of significance at 1%, 5% and 10% respectively			
[30] for sources for critical value			

*JB* Jarque–Bera normality test, *LM test* BG serial correlation LM test, *Het: ARCH* Heteroskedasticity test: ARCH, *RESET* Ramsey RESET, *D–W* Durbin–Watson statistics

^a,b^Indicate 1% and 5% level of significance

**Table 4:** FDI-outflow for symmetric and asymmetric ADRL bounds co-integration test

Table. ARDL bounds cointegration test results	Best ARDL model	F-statistic	Result
3. *F*_*Q*_ (ln*Q*|ln*K*, ln*L*, lnDum1, lnoutflow, $\ln \left( {{\text{outflow}}*{\text{Dum}}1} \right)$*,* lnPcrd, lnTrade, Brk)	ARDL(1, 2, 0, 2, 2, 2, 1, 2, 2)	9.4100^a^	Co-integration
Diagnostic test			
D–W	2.00387		
JB- normality test	2.0348	[0.36152]	
LM test	6.9903	[0.3550]	
Het: ARCH	17.278	[0.7478]	
RESET test	1.7286	[0.2179]	

Cusum & Cusumsq stable		I(0) Bound	I(1) Bound
Critical value bounds (*k* = 8)	10%	1.85	2.85
	5%	2.11	3.15
	1%	2.62	3.77
4. *F*_*Q*_ (ln*Q*|ln*K*, ln*L* lnDum1, lnoutflow^+^, lnoutflow^−^, ln*(*outflow*Dum1), lnPcrd, lnTrade, Brk)	ARDL(1, 0, 0, 2, 2, 2, 1, 1, 2, 2)	7.7703^a^	Cointegration
Diagnostic test			
D–W	2.1525		
JB- normality test	2.0348	[0.3615]	
LM test	14.161	[0.0885]	
Het: ARCH	17.278	[0.7478]	
RESET test	19.558	[0.6106]	

Cusum & Cusumsq stable		I(0) Bound	I(1) Bound
Critical value bounds (*k* = 9)	10%	1.8	2.8
	5%	2.04	2.08
	1%	2.5	3.68
The model selection ARDL is based on (AIC)			
a,b & c indicate level of significance at 1%, 5% and 10% respectively			
[30] for sources for critical value			

*JB* Jarque–Bera normality test, *LM test* BG serial correlation LM test, *Het: ARCH* Heteroskedasticity test: ARCH, *RESET* Ramsey RESET, *D–W* Durbin–Watson statistics

^a,b^Indicate 1% and 5% level of significance

### Impact of FDI-inflow- symmetric approach

In this section, our aim is tied to testing long-run association between FDI-growth nexus by incorporating the effect of financial crises, economic crises and COVID-19 pandemic and control variables using bounds test of the ARDL approach. The results culled from Tables 3 and 4 depict that the calculated *F*-statistic models 1–4 are 6.1032, 6.4246, 9.4100, and 7.7703, respectively, which supersedes the upper critical value at 5%. Therefore, the hypothesis stated in its null state reiterating no co-integration at 5% significant level for linear and nonlinear specifications are rejected. We proceed to estimate the models using the linear and NARDL bounds test approach. In spirit with the suggestion of Lütkepohl [27] for small sample size, we depended on AIC to ascertain the optimal lag length for the models which is pinned at lag 2.

The long- and short-run estimates for foreign direct investment (ln inflow) are reported in Table 5. The left side of Table 5 shows the estimated linear ARDL model, while the right side shows the estimated nonlinear ARDL model. Based on the linear ARDL model, the lag of ln*Q*(− 1) suggested positive relationship with current ln*Q*(− 1) at 5%. It indicates that past ln*Q*(− 1) is a significant determinant of current economic growth and this validates the adoption of dynamic model. FDI inflow has a positive and statistically significant association with economic growth both the long and short run at 5%. On average, a unit increase in FDI inflow will lead to 0.0209% and 0.0354% increase in both time periods. The positive effect in the FDI-growth link supports the FDI-led growth theory and in line with rising empirical entries in the case of Ehigiamusoe and Lean (2019) and Adegbite and Ayadi [2] in the case of Nigeria and Acquah and Muazu [1] evidence in the case of West Africa. As expected, the economic crisis encapsulating the financial, commodity crisis and COVID-19 pandemic exerts negative and significant impact on economic growth at 5% level both in the long- and short-run estimates. On the factor of production, gross fixed capital formation, labor and trade openness drive economic growth at 5%, both in the long and short-run and support the endogenous growth theory as reinforced by Paul Romer (1986) positing that some of the key variables in this study serve as the internal forces that drives economic growth. The coefficient of credit to private sector is positive and statistically insignificant at 10% level, in both time periods. A gleaned reason for this result is attributed to the underdeveloped nature of the financial system in Nigeria in mobilization and distribution of resources in the economy.

**Table 5:** symmetric and asymmetric effects of FDI-inflow and economic growth

Model Specification 1: F_Q_ (lnQ|lnK, lnL, Dum1, lninflow, $\ln \left( {{\text{inflow}}*{\text{Dum}}1} \right)$*,* lnPcrd, lnTrade, Brk)			Model Specification 2: F_Q_ (lnQ|lnK, lnL*,* Dum1, lninflow^+^, lninflow^−^, ln*(*inflow*Dum1), lnPcrd, lnTrade, Brk)		
Variable	Coefficient	Prob	Variable	Coefficient	Prob
*Part A: long-run estimate*					
*c*	4.1779	0.0017	*C*	0.3495	0.7379
ln*Q*(− 1)	0.0434a	0.072	ln*Q*(− 1)	− 0.1856	0.0776
lninflow	0.00209b	0.0238	lninflow^+^ (− 1)	− 0.0587a	0.0010
Dum1(− 1)	− 0.1942a	0.0012	lninflow^−^ (− 1)	0.0242b	0.0079
ln*K*	0.0365b	0.0423	Dum1(− 1)	− 0.1175b	0.0336
ln*L*(− 1)	2.3423a	0.0020	ln*K*(− 1)	0.7865a	0.0004
lnpcrd(− 1)	0.0717	0.1007	ln*L*(− 1)	0.5140	0.4119
lntrade(− 1)	0.0141	0.2873	lnPcrd(− 1)	0.3680a	0.0043
lninflow*dum1(− 1)	− 0.0728b	0.0043	lnTrade(− 1)	0.3638a	0.0007
Brk(− 1)	0.0665a	0.0159	lnFdi*Dum1(− 1)	− 0.0277b	0.0034
			Brk(− 1)	0.0282b	0.0387
*Part B: short-run estimate*					
∆ln(*Q*(− 1))	0.1515	0.2952	∆ln(gdp(− 1))	− 0.9572	0.0083
∆lninflow	0.0354b	0.0253	∆ln(inflow^+^)	− 0.0228a	0.0018
∆ln(dum1(− 1))	− 0.1771a	0.0003	∆ln(inflow^+^(− 1))	− 0.0417b	0.0130
∆ln(*L*)	0.4621b	0.0234	∆ln(inflow^−^)	0.0214b	0.0034
∆ln(pcrd)	0.0184	0.5654	∆ln(inflow^−^ (− 1))	0.0151b	0.0024
∆ln(trade)	0.0125	0.8878	∆(Dum1)	− 0.0781b	0.0024
∆ln(trade(− 1))	0.0284	0.1527	∆ln*K*	0.1499b	0.0426
∆ln(fdi*Dum1(− 1))	− 0.0518b	0.0102	∆ln(*L*)	1.1889b	0.0314
∆ln(brk(− 1))	− 0.0419a	0.0013	∆ln*(*pcrd)	0.1329b	0.0165
ecm(− 1)	0.1925a	0.0000	∆ln(trade)	0.0892a	0.0060
			∆ln(fdi*Dum1)	− 0.0207b	0.0109
			∆ln(brk)	0.0007	0.9531
			ecm(− 1)	− 0.1856a	0.0000
*Part C: Asymmetry result*					
Long run				53.531^a^	0.0000
Short run				0.1101^a^	0.0001

a and b Indicate 1% and 5% level of significance

On the impact of economic crisis (Dum1) to the FDI-growth nexus, the coefficients of the long- and short-run interaction terms (*ln*inflow*Dum1 and ∆*ln*(inflow*Dum1), which indicate whether economic crisis distorts or strengthens FDI, are negative in long and short run at 5%. These coefficients suggest that the negative long- and short-run effect of FDI on economic growth in Nigeria is further decreased during economic crisis in Nigeria. However, symmetric approach has some limitations: (1) linear model or symmetric approach can provide unrealistic or restrictive result which can lead to biased inference [25]. (2) asymmetric relationship between FDI-growth nexus amidst financial crises, economic crises and COVID-19 pandemic cannot be accounted for during recovery and recessionary period, hence, symmetric ARDL model cannot account for a robust negative or positive implications of FDI inflow on economic growth. (3) linear model cannot account for hidden co-integration in the model, hence, nonlinear would be a preferred model because it proffer solution to such econometric issues. The error correction model ${\text{ecm}} \left( { - 1} \right)$ takes an average of 19% to converge to long-run equilibrium in the next period.

### Impact of FDI-inflow-asymmetric approach

Based on space constraint, our discussion is based on the key variables. According to Table 5 (right side of the table), the asymmetric effect of FDI-inflow in Nigeria shows the positive and negative changes in FDI-inflow impact of economic growth are different during the series of economic crisis. At 1% alpha level, the long- and short-run parameter estimate of the positive shocks in FDI-inflow exerts negative influence on economic growth. Ceteris paribus, 1% positive shock in FDI-inflow depletes economic growth in Nigeria by 0.057% and 0.0417% in both time periods, respectively. The impact of economic crisis further aggravates the situation with the negative interaction term between (FDI-inflow-Dum1) in both short and long run at 5%. Furthermore, the long- and short-run coefficients of the negative shocks in FDI-inflow are positively linked to economic growth at 5%. Ceteris paribus, a single negative shock in FDI-inflow expands economic growth in Nigeria by 0.0242% and 0.0214% in both time periods, respectively. The impact of economic crisis decreases the positive interaction term between (FDI-inflow-Dum1) in both time periods at 5%. Comparatively, this study extends the previous submission by Jimborean and Kelber [24] who posited that the global financial crisis adversely affected growth and considers the moderating role of economic crisis on FDI-growth nexus. These findings are in line with rising claims that trade and financial flows remain the undisputable channel through which crisis transmits into the Nigerian economy [14] and supports the recent economic recession orchestrated by the commodity oil price shock in 2016 which led Nigeria to its first recession in decades, and a dip in the official price of crude oil adversely impacted by the COVID-19 pandemic in 2020.

**Fig. 1:**
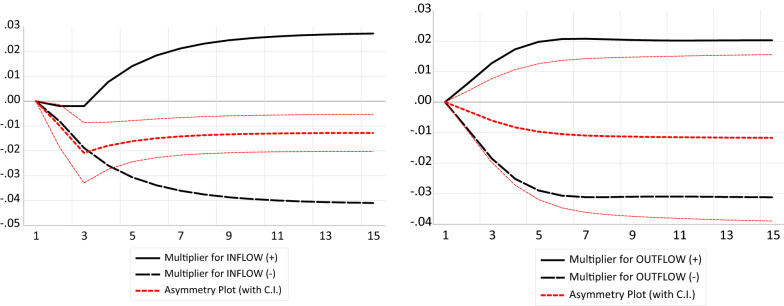
Multiplier graph for model specification 2, Multiplier graph for model specification 4

Capital, labor, domestic credit to private sector and trade openness exert beneficial and significant influence on economic growth in both time periods, respectively. Labor is insignificant in the long run. While the speed of adjustment converges to a long-run equilibrium on the average of 18.56%. For the asymmetric test, Wald test is used to test the equality of positive and negative shocks of FDI during economic crisis, and the result supports the long and short asymmetric, showing the magnitude (signs and coefficients) negative asymmetric is greater than positive asymmetric. Evidence is also shown in the dynamic multiplier graph of FDI (Fig. 1).

### Impact of FDI-outflow- symmetric and asymmetric approach

Table 6 presents the FDI-outflow for model specification 3 & 4 and the long- and short-run parameters of the linear and nonlinear ARDL models. Focusing on the linear model, the long- and short-run impact of FDI outflow is negative/positive and statistically significant at 5% level. On average, the coefficients show that a 1% increase in FDI-outflow (*ln*outflow) reduces economic growth by 0.0133% in the long run and increases economic growth by 0.0064% in the short run. This disparity between the long- and short-run impact on economic growth could be attributed to the complex nature of business cycle in explaining the nature and movement of FDI amidst financial crisis or other variants of crisis is succinct [8]. The impact of economic crisis on economic growth is negative and statistically significant in the long run at 5% level and positively linked with economic growth at 1% in the short run. Ceteris paribus, a 1% increase in economic crisis reduces economic growth by 0.0713% in the long run and increases economic growth by 0.1129% in the short run. The statistical relevance of FDI outflow either depletes or expands with the inclusion of economic crisis in the model. FDI-outflow exerts different impact with inclusion of the interaction term through various signs in both time periods. The interaction term (lnoutflow*Dum1) is positive and statistically in the long run, large enough to cushion the negative implication of FDI-outflow during the economic crisis, while the ∆ln(outflow*Dum1)-total effect of FDI outflow given economic crisis in the short run shows that the negative interaction is enough to dampen the positive impact of FDI output on economic growth.

**Table 6:** Symmetric and asymmetric effects of FDI-outflow and economic growth

Model Specification 3:F_Q_ (ln*Q*| lnoutflow, lngfcf, lnL, lnPcrd, lnTrade*,* lnKofgi, lnDum)			Model Specification 4:F_Q_ (lnrgdp| lnoutflow^+^, lninflow^−^, lnDum, lngfcf, lnL, lnPcrd, lnTrade, lnFDI*Dum, Brk)		
Variable	Coefficient	Prob	Variable	Coefficient	Prob
*Part B: long-run estimate*					
*c*	2.2352	0.0277	*c*	2.7030	0.0173
ln*Q*(− 1)	0.0521	0.442	ln*Q*(− 1)	− 0.0537	0.5566
lnoutflow(− 1)	− 0.0133b	0.048	lnoutflow^+^	− 0.0060b	0.0014
lnK	− 0.0875b	0.0437	lnoutflow-	0.0850b	0.0017
Dum1(− 1)	− 0.0713b	0.0153	ln*K*(− 1)	0.2209b	0.0088
ln*L*(− 1)	− 1.1816b	0.0253	Dum1(− 1)	0.0902	0.0151
lntrade(− 1)	0.0003	0.9839	ln*l*(− 1)	1.1432	0.0662
lnpcrd(− 1)	0.1737	0.0007	lntrade(− 1)	0.0115	0.6757
lnoutflow*Dum1	0.0291	0.5617	lnpcrd(− 1)	− 0.1555	0.0049
Brk(− 1)	0.0682	0.0053	lnoutflow*Dum(− 1)	0.0436	0.4257
		0.561	Brk(− 1)	0.0790	0.0042
*Part B: short-run estimate*					
∆ln(outflow(− 1))	0.0064b	0.0221	∆lnoutflow^+^	− 0.0323b	0.0114
∆(Dum1)	0.1129a	0.0002	∆lnoutflow-	0.0034b	0.0017
∆ln(*L*)	0.4191b	0.0466	∆ln*K*	0.0847b	0.0193
∆ln(trade(− 1))	0.0195b	0.0331	∆Dum1	0.2047b	0.0214
∆ln(Prcd)	0.0607a	0.0174	∆ln*L*	0.1791	0.6710
∆ln(outflow*Dum1)	− 0.0976b	0.0046	∆ln(trade)	0.0083b	0.0385
∆(Brk)	0.0124a	0.0003	∆ln(pcrd)	0.0425	0.1346
∆(Brk(− 1))	− 0.0469a	0.0001	∆ln(outflow*Dum1(− 1))	0.0535b	0.0093
ecm(− 1)	0.0521a	0.0000	∆(Brk)	0.0200b	0.0495
			∆(Brk(− 1))	− 0.0457b	0.0010
			ecm(− 1)	− 0.0537a	0.0000
*Part C: Asymmetry result*					
Long run				8.1566a	0.0135
Short run				4.4538a	0.0014

a and b Indicate 1% and 5% level of significance

Moving toward another contribution to the body of knowledge and with more emphasis on the key variables, we decomposed FDI-outflow into positive and negative shocks and account for its differential effect on economic growth during the multiple bouts of economic crisis in Nigeria, as seen in model specification (4) from Table 6. Examining positive shocks in FDI-outflow, the coefficients in the long and short run are negative and statistically significant at 5%. Ceteris paribus, a single positive shock in FDI-outflow depletes economic growth in long and short run in the case of Nigeria by 0.0060% and 0.03237%, respectively. The impact of economic crisis abates the situation with the positive interaction term between (FDI-inflow-Dum1) in both time periods at 5% level. Furthermore, the long- and short-run coefficients of the negative shock in FDI-outflow yield a beneficial association with economic growth and significant at 5%. On average, a unit negative shock in FDI-outflow increases economic growth in Nigeria by 0.0034% and 0.0850% in both time periods, respectively. The relationship between negative shocks in FDI-outflow and economic growth improves with the positive interaction term between (FDI-outflow-Dum1) in both short and long run at 5% significant level. Error correction mechanism remained statistically relevant and in line with the theoretical specification at 1% significant level. Finally, the asymmetric test is established by comparing the equality of positive and negative shocks of FDI outflows during economic crisis, and the result supports the long and short asymmetric showing the magnitude; negative asymmetric is greater than positive asymmetric in the long run, while the negative asymmetric is greater than positive asymmetric in the short run. Evidence is shown in the dynamic multiplier graph of FDI (Fig. 1).

## Summary and conclusion

This study evaluates the relationship between FDI and economic growth in Nigeria during the economic crisis (global financial crisis, commodity oil price shock, COVID-19 pandemic) by incorporating all factors of production (labor and capital) and some selected control variables under the framework of Cobb–Douglas production function. Employing data within the period of 1983–2020, this empirical adventure examined the transmission channels through which economic crisis could impact on economic growth in Nigeria vis-à-vis a positive or negative shocks of FDI inflow/outflows. Two models are considered in this study: (1) the linear ARDL model which is considered as the benchmark in the evaluation of symmetric effects of FDI on economic growth during the economic crisis in Nigeria. (2) Nonlinear ARDL model is decomposed FDI-inflow/outflow into positive and negative partial sum to investigate any possible asymmetric impact of FDI on economic growth through the moderating role of global financial crisis, commodity oil price shock, COVID-19 pandemic (dummy variable). By estimating and verifying the asymmetric effect of FDI, the spill-over (indirect) effects on economic growth in Nigeria can be ascertained under the auspice of negative shocks (a decline in FDI inflow/outflow) and positive shocks (a rise in FDI inflow/outflow). Using the nonlinear ARDL co-integration, the long-run association was confirmed in each of the model specifications between the economic growth, FDI-inflow/outflow, interaction term, labor, capital, financial development and trade openness.

The results from this empirical masterpiece are in twofold, in line the models implemented: (1) using linear ADRL model, the impact of FDI-inflow during the economic crisis has a negative effect on economic growth in both long and short run, while the impact of FDI-outflow is positively linked with economic growth in the long run. In the case of Nigeria, Federal Government should prioritize FDI inflow into target sectors with tremendous potential to create employment and serve as a source of foreign exchange through the auspices of knowledge sharing, investment in research and technological and infrastructural upgrade. However, in the event of divestment of FDI from Nigeria, sectors such as tourism, transport, manufacturing and agriculture are deemed self-sustainable, which, in turn, increases the domestic capacity of the Federal Republic of Nigeria. (2) On the nonlinear ARDL model, three forms of asymmetry in the association between FDI-inflow and economic growth were documented; the short-run asymmetry, long-run asymmetry and the adjustment asymmetry. Accordingly, we put on record that there is a significant difference between the positive and negative shocks/ innovations. The impact of short- and long-run asymmetry suggests that the positive shocks in FDI inflow have a negative (recessionary effect) on economic growth, and negative shock in FDI-inflow a positive (improvement on economic growth) on economic growth, while during economic crisis (the moderating role/interaction term) is positive which aggravates the situation under the positive shocks and further improves the situation under the negative shocks. The short- and long-run asymmetry indicate that the positive shock in FDI outflow has a reducing effect on economic growth, while the interaction term is positive and large enough to cushion the negative implication of FDI-outflow on economic growth during the economic crisis. The short- and long-run asymmetry indicate that the negative shock in FDI outflow has an increasing effect on economic growth, while the interaction term further improves economy with its positive effect during the economic crisis. The results spell hope and give rise to policy implications aimed at developing the financial system which is not yet at the acceptable threshold to engender growth compared with the stimulation effect of FDI in tranquility and crisis period. On the other hand, the FDI inflow remaining stable during crisis period may signal information differential or signal over-investment which increases the reliance of FDI by the central government of Nigeria as a culminator of resources to stimulate economic activities across sectors of the Nigerian economy. The germane advice is to apply rationing of FDI inflow and purposeful allocation to needy areas.

In the case of adjustment multiplier, the graphs show that the impulse response of economic growth to a negative variation in FDI-inflow/outflow is stronger than the positive variation; by intuition, a decrease in FDI as a result of adverse effect of economic crisis would have more devastating effect on economic growth than a rise in the FDI for the same level of magnitude. Further results from the control variables are Capital, labor, domestic credit to private sector and trade openness exerts positive and significant influence on economic growth in both the long and short run, respectively. Labor is insignificant in the long run. Further studies in this area should explore the role of domestic capacity and alternative sources of cushioning the negative effect of economic crisis in Nigeria.

## Data Availability

Yes, available on request.
